# Molecular detection of tick-borne pathogens in infected dogs associated with *Rhipicephalus sanguineus* tick infestation in Thailand

**DOI:** 10.14202/vetworld.2021.1631-1637

**Published:** 2021-06-23

**Authors:** Amornrat Juasook, Bunnada Siriporn, Natthaphat Nopphakhun, Pacharamol Phetpoang, Subongkoch Khamyang

**Affiliations:** 1 Bioveterinary Research Unit, Faculty of Veterinary Sciences, Mahasarakham University, Maha Sarakham, Thailand; 2 Faculty of Veterinary Sciences, Mahasarakham University, Maha Sarakham, Thailand

**Keywords:** dog, polymerase chain reaction, *Rhipicephalus sanguineus*, tick-borne pathogens

## Abstract

**Background and Aim:**

Tick-borne pathogens (TBPs) are of great concern having the potential to threaten canine health. Dogs infected with *Ehrlichia canis*, *Anaplasma platys*, *Babesia canis*, and *Hepatozoon canis* are commonly found in Thailand; *Rhipicephalus sanguineus* tick is the most common vector of diseases. This study aimed to determine the prevalence of common TBPs in dogs and their ticks in Thailand using polymerase chain reaction (PCR) and DNA sequencing methods.

**Materials and Methods:**

Forty-four blood samples were positively diagnosed with TBPs infection by microscopy. Samples were from animal hospitals in Maha Sarakham, Amnat Charoen, Nakhon Ratchasima, and Bangkok, Thailand, during January-June 2020. Five to six ticks were also taken from infected dogs, and then, both blood and tick were analyzed using PCR and DNA sequencing.

**Results:**

PCR results showed that *R. sanguineus* was the only tick species detected in this study. The appearance of single infection with *E. canis* was the most common infection found in dogs and ticks (64% and 82%, respectively). Correlation of pathogen infection in hosts and their vector was performed by similarity detection of pathogens between blood and tick samples based on PCR analysis in 29 samples (66%) but there was no significant differentiation.

**Conclusion:**

*E. canis* appears as the most common canine tick-borne pathogen in Thailand, which was detected in both healthy and sick dogs as well as in *R. sanguineus*. The findings show the relationships among host dogs, pathogens, and ticks. Veterinarians should be proactive in educating pet owners about the risks associated with ticks and their important pathogens and plan effective control strategies.

## Introduction

Tick-borne pathogens (TBPs) can cause critical infections that are potentially fatal. The incidence of tick-borne diseases has been reported to have increased worldwide in recent years. These diseases are of great concern because of their potential to threaten canine health and risk of the transmission of pathological agents from dogs to humans [1]. The brown dog tick, *Rhipicephalus sanguineus*, is the most common blood-sucking tick species found on dogs in tropical and subtropical regions [2,3], including in Thailand [4]. Four major canine TBPs have been reported in Thailand; *Ehrlichia canis*, *Anaplasma platys*, *Hepatozoon canis*, and *Babesia canis* [5-9]. Dogs are generally infected by the bite of infected ticks (*E. canis*, *A. platys*, and *B. canis*) or ingestion of infected ticks (*H. canis*) [10]. Symptoms in infected dogs vary in their effects from asymptomatic to severe. The most common clinical signs are weight loss, fever, anorexia, and hemolytic anemia [11]. It has been reported that one tick species can be a vector for various pathogens [12] and can transmit those pathogens concurrently in a single blood meal [13]. Thus, if the host was infected with two or more of these agents, it could result in more complicated pathogenicity and poor prognosis [14,15]. Diagnosis of canine tick-borne diseases should select appropriate methods, including history of infestation with ticks, physical examination, compatible clinical manifestation, and laboratory confirmation with hematology and serology [16].

Awareness of the occurrence and distribution of TBPs in dogs, as well as in their vectors, is critical for effective control. Nowadays, molecular techniques to confirm blood parasite infection by polymerase chain reaction (PCR) are widely used because they have high sensitivity and specificity [4,5,17-21]. There are several reports on epidemiological surveys of TBPs in Thailand using PCR technique that has revealed an increased incidence in recent years [4,5,17]. However, most veterinary hospitals in Thailand diagnose TBPs by microscopic examination of peripheral blood smears, while less commonly, serology using test kits and other molecular techniques are used.

Epidemiological surveys of ticks and their transmitted pathogens in dogs in Thailand are lacking. This study aimed to determine the prevalence and verify the host and vector relationships of TBPs in dogs and their ectoparasite using molecular methods to provide a better understanding of the situation of canine TBPs in Thailand.

## Materials and Methods

### Ethical approval

This study was approved by the Institution Animal Care and Use Committee of Maha Sarakham University, Thailand (IACUC-MSU-050/2019).

### Study period and location

The study was conducted from January to June 2020. All samples were obtained from animal hospitals in Maha Sarakham, Amnat Charoen, Nakhon Ratchasima, and Bangkok, Thailand. The study was conducted at Molecular Laboratory, Faculty of Veterinary Sciences, Mahasarakham University, Thailand.

### Blood sample collections

Blood from 44 dogs was obtained from veterinary clinics and hospitals in Thailand. The collection criteria for the dog blood samples were as follows: (i) check both healthy and sick dogs for the presence of tick-borne infection (*E. canis*, *A. platys*, *B. canis*, or *H. canis*) which the veterinarian diagnosed using the blood smear method and (ii) check for an existing tick infestation. After TBPs were detected, the remaining blood samples of at least 0.5 mL in a sterile EDTA coated tube were stored in a freezer (−20°C) in the clinics or hospitals until extraction of DNA in the laboratory.

### Tick collection and identification

Samples of ticks were collected from each dog if the blood test was a positive test for TBPs. Dogs were individually inspected for 10 min each [13]. All developmental stages (i.e. larval, nymph, and adult) and gender (female and male) ticks found were manually detached and pooled into labeled tubes, individualized per dog, containing 70% ethanol [22]. Ticks samples were kept at −20°C until DNA extraction and were identified using morphological keys [23].

### Blood and tick DNA extraction

Following morphological identification, 5-6 ticks from each infested dog were processed for the extraction of pathogen DNA. All blood and tick DNA samples were extracted using GF-1 Blood DNA Extraction Kits and GF-1 Tissue DNA Extraction Kits (Vivantis^®^, Malaysia), respectively, following manufacturer’s protocol, and stored at −20°C until further use. Before the DNA extraction, ticks were removed from 70% alcohol, washed in phosphate-buffered saline (PBS), and air-dried on tissue paper for 10 min following the protocol of Zaid *et al*. [19] to remove microorganisms on the surface of the ticks before DNA extraction. Then, ticks were separately sliced into small pieces using a sterile scalpel depending on their size according to the protocol of Geurden *et al*. [22]. Briefly, ticks were cut in half in a mediosagittal direction leaving the salivary glands intact. For each tick sample, a new sterile blade was used to avoid possible contamination between samples. If fully fed, ticks were cut again into two and only the parts with salivary glands were transferred into a labeled 1.5 mL tube containing 100 mL PBS. The other parts of the tick were sliced to smaller pieces using sterilized scissors and pooled in the tube for further DNA extraction processing.

### PCR amplification and sequencing

PCR technology was used for the detection of TBPs in dog and tick DNA samples, followed by DNA sequencing for precise determination of pathogens. Before the detection of pathogens in the tick samples, the mitochondrial *16S* rRNA (mt-rrs) was detected from tick DNA samples to confirm the identification of the tick as *R. sanguineus*. After positive confirmation of the control genes, conventional PCR targeting the TBPs was performed. The target genes in blood and tick samples, primers, PCR conditions, and references for each pathogen are provided in Table-1 [4,20,24,25]. PCR mixtures (OnePCR^®^, GeneDirex, Taiwan) were prepared following the manufacturer’s recommendation, then, PCR reactions were run in a ProFlex PCR System (Applied Biosystems, USA). PCR products were subjected to electrophoresis in 1% agarose gel and virtualized with a UV lamp (UVITEC, United Kingdom).

**Table-1:** Primers, target genes, and polymerase chain reaction conditions used in the detection of various TBPs and control gene in blood and tick samples.

Pathogen	Target	Primer sequence (5’-3’)	Annealing temperature (°C)	Product size (bp)	Reference
*E. canis*	16S rRNA	5’-CCATAAGCATAGCTGATAACCCTGTTACAA-3’ 5’-TGGATAATAAAACCGTACTATGTATGCTAG-3’	57	380	[24]
*A. platys*	P44	5’- GCTAAGTGGAGCGGTGGCGATGACAG-3’ 5’- CGATCTCCGCCGC TTTCGTATTCTTC-3’	62	520	[20]
*B. canis*	18S rRNA	5’-CAGGGCTAATGTCTTGTAATTGG-3’ 5’-ATTTCTCTCAAGCTCCTGAAGG -3’	62	557	[24]
*H. canis*	18S rRNA	5’-ATACATGAGCAAAATCTCAAC 5’-CTTATTATTCCATGCTGCAG	62	666	[4]
Tick control	mt-rrs	5’-TGCTCAATGATTTTTTAAATTGCTGTGG-3’ 5’-CCGGTCTGAACTCAGATCAAGTA-3’	56	460	[25]

*E. canis=Ehrlichia canis*, *A. platys=Anaplasma platys*, *B. canis=Babesia canis*, and *H. canis=Hepatozoon canis*

For sequence analysis, PCR products were randomly selected from the positive blood and tick samples, then sent for nucleotide sequencing by ATGC CO., LTD. Company (Pathum Thani, Thailand) using Sanger’s sequencing method to confirm the identity of the amplified fragment. Sequence data were compared to previous reports using the Basic Local Alignment Search Tool (BLAST) of the U.S. National Center for Biotechnology (https://blast.ncbi.nlm.nih.gov/Blast.cgi). All of derived sequences were used to construct a phylogenic tree employing the neighbor-joining method (MEGA X: https://www.megasoftware.net/) [26].

### Statistical analysis

The occurrence of TBPs infections in dog and tick was analyzed using Chi-square tests at a 95% confidence interval (p=0.05), using IBM SPSS Statistics 20.0 software (IBMCorp., NY, USA).

## Results

### Clinical data

All sick dogs had common clinical signs of canine blood parasite diseases such as lethargy, emaciation, fever, or pale mucous membranes, and were diagnosed as positive using microscopic methods. In addition, healthy dogs that were brought to a veterinarian for a general health check, and had no obvious clinical signs but were positive for TBPs were included in the study. Most (57%) of the sampled dogs were male, while the remaining 43% were female. The microscopic examination of the blood showed that the majority of both healthy and sick dogs were coinfected with two pathogens (61%) while single infections (39%) were less frequent. Single infections with *E. canis* appeared in 10 (23%) dogs followed by *A. platys* in 7 (16%) dogs. Coinfections with *E. canis* and *A. platys* were most prevalent (54%) followed by *E. canis* and *H. canis* (7%). At the time of blood collection, 5-6 ticks were randomly collected from each infested dog for DNA extraction and identification of pathogens. All ticks found in this study were morphologically identified to be *R. sanguineus*.

### PCR analysis of TBPs in blood and tick DNA samples

**Figure-1:**
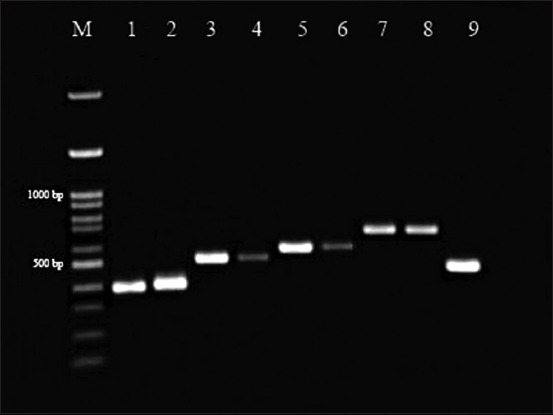
Polymerase chain reaction electrophoresis amplicons sample of tick-borne pathogens. Lanes 1 and 2 represented *Ehrlichia canis* amplicons of blood and tick samples at 380 bp, respectively. Lanes 3 and 4 represented *Anaplasma platys* amplicons of blood and tick samples at 520 bp, respectively. Lanes 5 and 6 represented *Babesia canis* amplicons of blood and tick samples at 557 bp, respectively. Lanes 7 and 8 represented *Hepatozoon canis* amplicons of blood and tick samples at 660 bp, respectively. Lane 9 represented *Rhipicephalus sanguineus* amplicons at 460 bp. M; marker, bp; base pair.

TBPs in blood and tick DNA samples were amplified using conventional PCR technique; PCR results for each pathogen amplicon in blood and tick samples is shown in Figure-1. The amplicons were then sent for sequencing and used to identify TBPs based on their DNA sequences. From a total of 44 dog blood samples, single infections occurred in 28 (64%) of dogs, while coinfection with more than one pathogen occurred in 16 (36%) of the dogs examined. Single infection was found only with *E. canis* pathogens, while coinfections most frequently involved *E. canis* and *A. platys* (14%) followed by *E. canis* and *B. canis, E. canis* and *H. canis* (11% and 4%, respectively). Using PCR, infections with more than two pathogens were observed in 3 (7%) dogs which were undetectable by microscopic methods. In tick DNA samples, 82% (n=36) of the ticks were positive for a single infection with *E. canis*, while coinfection with two pathogens was found in 5 (11%) of pooled tick samples and three mixed pathogens infections were found in 3 (7%) pooled tick samples (Table-2).

**Table-2:** Microscopic and molecular detection of TBPs in blood and tick DNA samples

TBPs detected	No. of dogs (%)			p-value

	Microscopic detection	Blood DNA detection	Tick DNA detection	
1 TBP only	17 (39)	28 (64)	36 (82)	-
*E. canis*	10 (23)	28 (64)	36 (82)	
*A. platys*	7 (16)	0 (0)	0 (0)	
*B. canis*	0 (0)	0 (0)	0 (0)	
*H. canis*	0 (0)	0 (0)	0 (0)	
2 TBPs	27 (61)	13 (29)	5 (11)	0.123^*^
*E. canis* and *A. platys*	24 (54)	6 (14)	2 (4)	
*E. canis* and *B. canis*	0 (0)	5 (11)	0 (0)	
*E. canis* and *H. canis*	3 (7)	2 (4)	3 (7)	
*A. platys* and *B. canis*	0 (0)	0 (0)	0 (0)	
*A. platys* and *H. canis*	0 (0)	0 (0)	0 (0)	
*B. canis* and *H. canis*	0 (0)	0 (0)	0 (0)	
>2 TBPs	0 (0)	3 (7)	3 (7)	0.368^*^
*E. canis, A. platys,* and *B. canis*	0 (0)	2 (4)	2 (4)	
*E. canis, A. platys, and H. canis*	0 (0)	0 (0)	1 (3)	
*A. platys, B. canis,* and *H. canis*	0 (0)	0 (0)	0 (0)	
*E. canis, A. platys, B. canis,* and *H. canis*	0 (0)	1 (3)	0 (0)	
Total number of dogs	44 (100)	44 (100)	44 (100)	

^a^Percentages were calculated based on the total number of blood and tick samples.

* p>0.05 (comparison of percentage of detected TBPs in blood DNA and tick DNA using PCR analysis). TBPs=Tick-borne pathogens, *E. canis=Ehrlichia canis*, *A. platys=Anaplasma platys*, *B. canis=Babesia canis*, and *H. canis=Hepatozoon canis*

### Association of TBPs in dogs and their vector

The presence of pathogen DNA was compared in blood and ticks from the same dog to evaluate host and vector relationships. All of 44 (100%) blood and tick pools were both positive for *E. canis*. Of these 29 (66%), the same pathogens that presented in both dog blood and tick samples were detected using PCR analysis discovered only infections of *E. canis* (25/44), coinfections of *E. canis* and *A. platys* (3/44) and coinfection of *E. canis, A. platys*, and *B. canis* (1/44).

The DNA of different pathogens in blood and tick samples was detected in 15 of 44 (34%) dogs. The DNA of some pathogens (*A. platys, B. canis*, and *H. canis*) was detected in blood from 12 dogs but not in their tick, while the DNA of some pathogens (*B. canis* and *H. canis*) was detected in ticks removed from three dogs whose blood samples were negative for those pathogens. However, there was no significant difference between the detection of pathogen DNA in dogs’ blood and their ticks (p>0.05).

### DNA sequencing analysis

**Figure-2:**
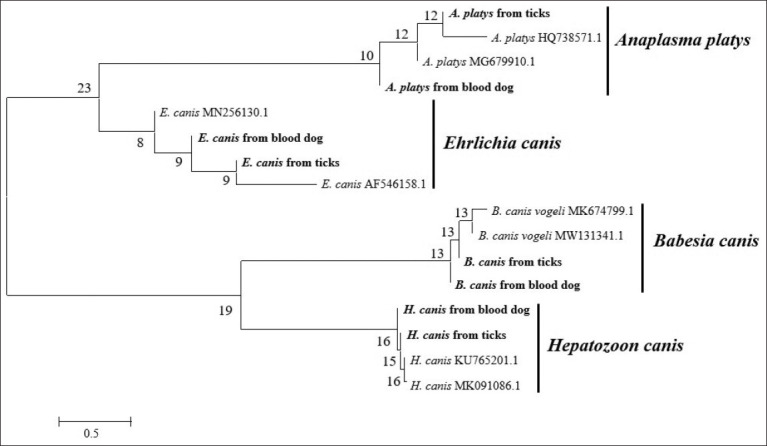
Phylogenetic tree of sequences obtained from dog blood and tick samples in this study (indicated in bold typeface) together with eight related sequences in GenBank. Sequences were compared with the neighbor-joining method operated by MEGA X. The percentage of trees in which associated taxa clustered together is shown next to the branches.

For each genus of detected TBPs and tick species confirmation, duplicate positive amplicons were subjected to sequencing and BLAST analysis. The detection of mt-rrs from ticks showed that all shared 99.76% identity with reported mt-rrs sequences of *R. sanguineus* (GenBank: MF351574.1). All obtained sequences for each pathogen were found to share 99-100% identity, including *E. canis* in both blood and tick samples showed 100% identity (GenBank: MN256130.1); *A. platys* in both blood and tick samples showed 99.29% identity (GenBank: MG679910.1); *B. canis* in blood and tick samples showed 99.41% and 99.23% identity (GenBank: HM590440.1), respectively, and *H. canis* in blood and tick samples showed 99.21 and 100% identity (GenBank: KC138532.2), respectively. A phylogenic tree constructed from all obtained sequences with related sequences in GenBank is presented in Figure-2.

## Discussion

This present study showed that *E. canis* was the most detected pathogen in blood and tick samples and this finding correlated with microscopic diagnoses. The high detection rate of *E. canis* obtained in this study is similar to that in the previous reports in Southeast Asian countries[21,27], including in Thailand [5,17].

*E. canis* is a common pathogenic rickettsia of domestic dogs worldwide. The brown dog tick, *R. sanguineus*, acts as the vector of *E. canis* and causes canine monocytic ehrlichiosis [28]. The disease results in variable non-specific clinical manifestations and is mainly seen in three stages: Acute, subclinical, and chronic stages. Clinical signs commonly present with fever, lethargy, anemia, anorexia, weakness, epistaxis, and lymphadenomegaly [29]. Moreover, human monocytic ehrlichiosis has also been reported as zoonotic, in which *E. canis* DNA was discovered by PCR from human cases [30]. Therefore, our study suggested that canine monocytic ehrlichiosis is the most common canine blood parasite disease in Thailand that can be found in subclinical infected dogs and should remind us of the potential zoonotic risk to humans.

Concurrent infections with more than 1 TBPs were observed in dog blood in this study. The appearance of coinfection in the infected animal may possibly exacerbate pathogenicity and complications [13,14]. Therefore, a better understanding of the pathogenesis and the progression of clinical, hematological, and biochemical abnormalities of canine tick-borne disease will be important in choosing appropriate diagnostic methods and in establishing the best strategies for treatment and control.

The current study revealed that false-positive results of a single infection of *A. platys* were as high as 16% detected by microscopic methods, but none were detected by PCR technique. *A. platys* is the causative agent of infectious canine cyclic thrombocytopenia usually found in dogs and *R. sanguineus* ticks which was reported to be a common vector of *A. platys* [31,32]. The most common diagnostic method is morulae identification in stained blood smears, although observing this pathogen as inclusion bodies inside the platelets of infected dogs is known to be difficult [33]. Inclusion bodies in platelets can be associated to other diseases as platelets play an important role during the inflammatory response [34]. These cytoplasmic inclusion bodies are identified by the formation of granules in the center region of the platelet as false nuclei, resembling morulae of *A. platys* agent [35]. A previous report also revealed that one out of every six infected dogs had platelet inclusions and about 5% of the platelets had inclusions [36]. Moreover, false nuclei of the platelet have also been reported in *A. platys* diagnosis in dogs [37]. Therefore, the diagnosis of *A. platys* needs to differentiate unspecific platelet inclusions and molecular techniques are necessary for the confirmation to avoid misdiagnosis and excessive treatment.

In this study, ticks were collected from infested dogs at the time of presentation to verify the host and vector relationship. All tick pools (5-6 ticks/dog) from 44 positive dogs were *R. sanguineus*, which is the most common ectoparasite found on domestic dogs in Thailand [4]. TBPs detection in *R. sanguineus* tick samples revealed that *E. canis* had the highest rate of infection which correlated with the result in the dog blood samples. The pathogens detected in both host blood and their ticks might be caused by blood-sucking vectors having taken up the infection from the host while feeding on them and they can transmit the pathogens to further hosts during their next feeding by transstadial transmission. In addition, the coinfections in the ticks that were detected in this study might be due to the ability of ticks to harbor various microorganisms since they feed on several hosts during their life stages [38].

Interestingly, three tick pool samples were positive for *B. canis* and *H. canis* which were not detected in any of their host blood samples. *R. sanguineus* is a three-host tick [23], which requires three hosts throughout its life cycle; thus, pathogens detected from the ticks might have been obtained from the previous hosts and not from the present dog. A previous study also provided evidence for transovarial transmission of *Babesia* spp. in dog tick that can cause canine babesiosis [39]. Furthermore, they might transmit these pathogens to present or other hosts during their life cycle. Hence, these infected ticks act as a potential vector that can transmit the pathogens to their present host or another dog population. Tick-borne protozoal and bacterial hemoparasites of veterinary importance in ticks also have been reported in Thailand [38,40-41]. Therefore, detection of pathogens in tick vectors is essential to demonstrate the potential for disease in particular endemic areas. In this study, the sequencing results revealed the similarities of pathogens identified from the host and those identified in ticks removed from them, and also showed 99-100% identity with previous reports in GenBank by BLAST analysis. Although the statistical analysis did not reveal significant differences between the detection of pathogen infection in dogs and ticks, the results of this study still support the relationship among ticks, pathogens, and dogs.

## Conclusion

Our result reveals that TBPs in dogs and infestation with *R. sanguineus* ticks are highly prevalent, although some dogs in this study had no significant symptoms of infection, *E. canis* being the most prevalent pathogen in dog and their tick. Therefore, we should increase awareness among dog owners regarding the importance of controlling ticks and their transmitted pathogens. Furthermore, other tick-borne zoonotic pathogens should be further investigated. Further studies are needed to estimate the impacts to local residents and animal husbandry by these vectors and pathogens and to establish effective measures to control the vector ticks.

## Authors’ Contributions

AJ and BS: Designed the study. AJ, BS, NN, PP, and SK: Coordinated sample collection and performed the experiments. AJ, BS, NN, PP, and SK: Analyzed the results. AJ: Drafted the manuscript. All authors read and approved the final manuscript.
